# Management and Follow-Up of a Case of Gestational Gigantomastia in a Brazilian Hospital

**DOI:** 10.1155/2014/610363

**Published:** 2014-08-04

**Authors:** Pollyana Eler dos Reis, Natalia Quarto Blunck Santos, Fernanda Alves Barbosa Pagio, Fabio Chambô, Danielle Chambô, Antônio Chambô Filho

**Affiliations:** ^1^Department of Obstetrics and Gynecology, Hospital Santa Casa de Misericórdia, 29025-023 Vitória, ES, Brazil; ^2^Department of Plastic Surgery, Hospital Santa Casa de Misericórdia, 29025-023 Vitória, ES, Brazil; ^3^Department of Mastology, Hospital Santa Casa de Misericórdia, 29025-023 Vitória, ES, Brazil

## Abstract

Gigantomastia is a breast disorder that is associated with an exaggerated, rapid growth of the breasts, generally bilaterally. Since the pathology is rare and has seldom been described, its etiology has yet to be fully established, although there are speculations that a hormonal component may play an important role. Treatment is aimed at improving the clinical and psychological symptoms; however, the best therapeutic option varies from case to case. The present report describes a case of gestational gigantomastia seen at the Department of Obstetrics and Gynecology of the *Hospital da Santa Casa de Misericórdia*, Vitória, Espírito Santo, Brazil, in a primigravida in the second trimester of pregnancy. The report follows this patient from her diagnosis until the completion of treatment with a third and final surgical procedure.

## 1. Introduction

Gestational hypertrophy of the breast or gestational gigantomastia is a rare clinical condition of unknown etiology, characterized by exacerbated, incapacitating breast growth during pregnancy, with physical and psychological complications that directly affect the patient's quality of life and the progression of her pregnancy [1]. Although its etiology has yet to be clarified, it has been associated with the response of breast receptors to gestational hormones and with hyperprolactinemia [2–4]. Gestational gigantomastia was first reported by Palmuth in 1648 and since then fewer than 100 cases have been reported in the literature [5].

Because of the anatomical damage resulting from the rapid, exaggerated breast growth, ulcerations, infections, and even areas of local necrosis may occur, requiring palliative procedures aimed at improving the patient's comfort throughout her pregnancy [6]. Nevertheless, choosing the optimal therapeutic procedure to be implemented depends on the severity of each case [7, 8]. Due to the high rates of recurrence of the disease in subsequent pregnancies, simple mastectomies rather than reduction mammoplasties are generally performed, with the aim of removing all the mammary glands, thus protecting future pregnancies [9].

The objective of this paper is to describe a case that occurred in a primigravida diagnosed with the disorder in the second trimester of pregnancy, relating the approach adopted by the team at the Department of Obstetrics and Gynecology of the* Santa Casa de Misericórdia* Hospital, Vitória, Espírito Santo, Brazil.

All the procedures, including the present report, were conducted in compliance with the appropriate ethical principles, with approval by the internal review board of the* Escola Superior de Ciências* of the* Santa Casa de Misericórida*, Vitória, Espírito Santo, Brazil (CEP/EMESCAM), under reference number 12881813.0.0000.5065.

## 2. Case Presentation

DMM, a white, 24-year old married primigravida, was referred to the Department of Obstetrics and Gynecology of the* Santa Casa de Misericórdia* Hospital in Vitória in the 22nd week of pregnancy, complaining of bilateral breast growth and intense mastalgia, and reporting fever, although her temperature had not been taken. There was periareolar bleeding on her left breast.

Clinical examination showed voluminous breasts with dark skin and diffusely hyperemic areas, with tissue infiltration producing a hardened consistency, suggestive of a localized infection in both breasts. A ulcer was present on the lower outer quadrant of the right breast and an actively bleeding periareolar ulcer with necrotic tissue on the left breast. The patient was treated with antibiotics for the infection. Treatment with cabergoline and corticosteroids, initiated when the patient was first admitted to hospital at a different institute, was maintained for a further 10 days. No improvement was seen in the patient's clinical condition and the disease progressed with a substantial increase in the volume of her breasts. As a result of this exaggerated increase, the patient began to have difficulty moving around and had episodes of intense bleeding from the sores on her breasts resulting from local necrosis. Histology of biopsied material showed periductal fibrosis in the right breast and adenosis in the left breast, in both cases the results being suggestive of gestational macromastia.

**Figure 1:**
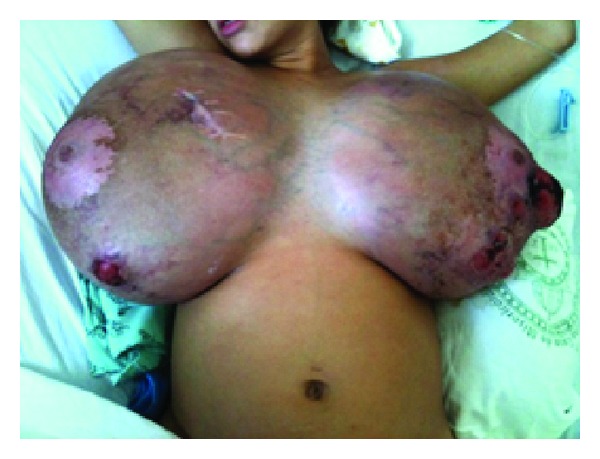
Patient in the 28th week of pregnancy. Note the excessive breast volume that prevented her from moving around or changing position in bed, causing extreme distress.

On the 41st day of hospitalization, the patient developed dyspnea, malaise, and generalized anxiety disorder. This condition was a consequence of the excess breast volume, which kept her confined to bed, provoking joint pain, particularly in her spine, resulting from the dorsal decubitus position that she was obliged to adopt due to the weight of her breasts, which prevented her from walking or even changing position in bed (Figure 1). Because of her distress, it was decided to interrupt her pregnancy and perform a Cesarean section at 28 weeks and 4 days of pregnancy following corticosteroid therapy to mature the fetal lungs. Surgery proceeded uneventfully and a live baby girl was born weighing 1,200 grams, with Apgar scores of 6 and 9 at one and five minutes, respectively. The infant was admitted to the neonatal intensive care unit where she remained for 99 days. At her release from hospital she weighed 3,160 grams. Her mother made a good recovery following the Cesarean section. She was treated with bromocriptine 5 mg/day and cabergoline 1 mg/day; however, there was no improvement in the size of her breasts. Some days later, a further course of antibiotics was initiated to treat an infected sore on the left breast, from which* Pseudomonas aeruginosa* and extended-spectrum *β*-lactamase producing* Klebsiella pneumoniae* (ESBL-KP) were identified on culture.

On the 53rd day after her Cesarean section, the patient developed sparse pustules with hyaline secretion and reddish borders on her trunk, neck, and lower limbs, suggestive of skin candidiasis, and antifungal treatment was initiated. Because of this secondary skin infection resulting from the gigantomastia, the patient remained in hospital for 108 consecutive days, only being released 64 days after her Cesarean section.

After the infections were under control, the patient was submitted to simple bilateral mastectomy, which proceeded without any complications. One year later, bilateral breast reconstruction was initiated, with the use of myocutaneous flaps from the serratus anterior muscle and the pectoralis major muscle and insertion of a 300 mL SILIMED tissue expander. During a second surgical procedure, the expander was exchanged for the definitive silicone breast implant, allowing equalization to improve the symmetry of the reconstruction.

**Figure 2:**
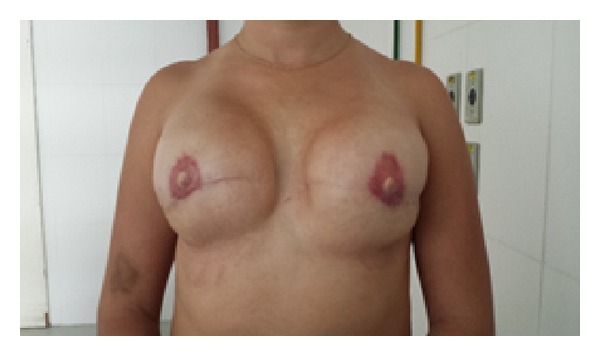
Frontal view showing the outcome of the third and final plastic surgery procedure in which the nipple-areola complex was reconstructed bilaterally.

During a third and final plastic surgery procedure, the nipple-areola complex was reconstructed bilaterally, using grafting and a local flap (Figure 2).

The patient progressed satisfactorily following each surgical procedure performed and she is currently in good health, both from a clinical and psychological point of view.

## 3. Discussion

Gestational gigantomastia is a complication whose etiology and pathogenesis have yet to be fully clarified; however, it has been speculated that placental hormones may trigger the condition [10–12]. This exaggerated increase in breast volume occurs most commonly at the end of the first trimester of pregnancy, coinciding with the period of peak gonadotropin production, thus giving strength to the hypothesis of a hormonal association [4]. There is no association between this excessive breast growth and the number of pregnancies the patient has had, although the condition is more common in multiparas [2, 13–19]. In the current case, the patient was a primigravida who was admitted to hospital at 22 weeks of pregnancy, although her breasts initially began to grow in size in the 15th week of pregnancy.

As a result of the substantial increase in the size of the breasts, physical consequences develop, including postural problems, skin ulcerations, and localized bleeding, in addition to the effect of the condition on the patient's psychological status, directly affecting her pregnancy [2, 5, 20]. The patient described in the present report developed depression and her maternal risk factor increased, since, with the progressive increase in the size of her breasts, she developed necrosis and recurrent infections to such an extent that her pregnancy had to be interrupted at 28 weeks of gestational age.

Gestational macromastia is a clinical condition that, theoretically, requires surgical intervention, since it is a phenomenon that could be triggered again by subsequent pregnancies, even those resulting in abortion [5, 13, 19].

In general, pathology of the surgical specimens shows glandular hyperplasia with an increase in the connective tissue and no involvement of the adipose tissue. Other findings include dilation of the lymphatic ducts, fibroadenomatosis, and cystic formation [10, 13, 14, 21]. In the present case, pathology revealed breast tissue with fibrocystic breast disease and ductal hyperplasia without atypia.

With the introduction of dopaminergic receptor agonists, other less aggressive forms of management began to be adopted with good results, irrespective of when the medication was initiated and despite the fact that these drugs neither avoided nor minimized the complications of the condition such as ulcerations and local necrosis [5, 14–17]. In the present case, both bromocriptine and cabergoline were used, both at the recommended doses; however, there was no improvement in the progression of the pathology.

The objective of the palliative treatment given for gestational gigantomastia is to improve the adverse conditions affecting pregnancy, principally by minimizing pain and providing psychological support. Definitive treatment for the condition will vary from case to case; however, simple mastectomy remains the best therapeutic alternative, since with this procedure, the entire mammary gland including the nipple-areola complex is removed. In the case in question, analgesics were used continuously, dressings were changed daily on the areas of ulceration and necrosis, and antibiotics were given. With respect to the surgical treatment, the decision was made to perform a simple mastectomy due to the fact that the patient was young and wanted to become pregnant again in the future [22].

In general, three surgical procedures are required to perform breast reconstruction, with an interval of approximately six months between one procedure and another in order to assure optimal adaptation and breast symmetry. In the case reported here, the healthcare team complied with the recommended time interval between the surgical procedures, with the entire treatment taking approximately two years to complete. The final result was considered satisfactory.

In conclusion, although gestational gigantomastia is a rare condition, its early diagnosis is of extreme importance in enabling the optimal pharmaceutical and/or surgical treatment to be implemented immediately. In the present case, pharmacotherapy failed to alter the clinical progression of the condition and the definitive treatment selected was a simple mastectomy with the objective of removing all the mammary glands. Considering the young age of the patient in question, the complete removal of these glands does prevent a recurrence of the condition in case of a future pregnancy.
